# Acute Esophageal Necrosis in a Patient With Prostate Cancer Postchemotherapy

**DOI:** 10.14309/crj.0000000000000366

**Published:** 2020-04-07

**Authors:** Eric Grisham, Suha Abu Khalaf, Vanessa Kuwajima

**Affiliations:** 1 Department of Internal Medicine, University of Missouri Columbia, Columbia, MO; 2 Department of Gastroenterology, University of Missouri Columbia, Columbia, MO

## Abstract

Acute esophageal necrosis (AEN) describes a potentially irreversible injury to esophageal mucosa secondary to vascular hypoperfusion. An 84-year-old man was admitted for the correction of a displaced nephrostomy tube and management of acute kidney injury. During his stay, the patient developed odynophagia and acute gastrointestinal hemorrhage. Despite mild initial symptoms, diffuse circumferential black esophageal mucosa was visualized on endoscopy, and a diagnosis of AEN was made. This unique case highlights the association between AEN and leukopenia, vascular disease, hypercoagulability, and infection. Presentations such as this patient should prompt the physicians' consideration of this differential earlier.

## INTRODUCTION

Acute esophageal necrosis (AEN), also known as black esophagus or Gurvits syndrome, is a rare, multifactorial injury to the esophageal mucosa, which carries a high mortality rate. AEN typically occurs in men and has an estimated prevalence overall of 0.001%–0.28% reported in various studies.^1,2^ The most well-supported theory explaining the pathophysiology of AEN describes a “two-hit” hypothesis, consisting of a low-flow vascular event that predisposes the esophageal mucosa and possibly the submucosa to caustic injury secondary to exposure to reflux of acid and pepsin.^1^ We report a case of AEN in an 84-year-old man with prostate carcinoma whose early symptoms were odynophagia and decreased oral intake.

## CASE REPORT

An 84-year-old man was admitted for the correction of a displaced nephrostomy tube and management of acute kidney injury. His medical history was significant for stage IV prostate carcinoma undergoing chemotherapy with docetaxel, bilateral obstructive hydronephrosis, gastroesophageal reflux disease, peptic ulcer disease, hypertension, hyperlipidemia, and ischemic cardiomyopathy. Initial laboratory test result values included white blood cells (WBCs) 11.62 × 10^9^/L, hemoglobin 9.9 g/dL, hematocrit 31.0%, platelets 88 × 10^9^/L, serum glucose 95 mg/dL; creatinine 4.35 mg/dL (baseline 2.92 mg/dL), potassium 5.4 mmol/L, blood urea nitrogen 76 mg/dL, anion gap 24 mmol/L, and alkaline phosphatase 319 U/L. The day after admission, compression doppler ultrasonography revealed a proximal subacute/chronic deep vein thrombosis, and he was found to have a vesicocutaneous fistula infection on suprapubic ultrasound. The patient received coumadin therapy with a heparin bridge, and meropenem which was deescalated to ciprofloxacin.

On day 5, the patient's WBC count dropped to 0.65 × 10^9^/L with a neutrophil count of 0.31 × 10^9^/L, likely a sequela of chemotherapy given 9 days before his admission. His other laboratory test results were significant for the following: hemoglobin 9.2 g/dL, hematocrit 27.7%, platelets 78 × 10^9^/L; creatinine 3.67 mg/dL, potassium 3.9 mmol/L, blood urea nitrogen 83 mg/dL, anion gap 18 mmol/L, alkaline phosphatase 158 U/L, prothrombin time 31.1, international normalized ratio (INR) 2.7, and blood smear was significant for elliptocytes, Burr cells, anisocytosis, and rare schistocytes. Hematology/oncology was consulted for neutropenia, and he was prescribed granulocyte-colony stimulating factor with complete resolution of his leukopenia after 2 doses because WBCs increased to 15 × 10^9^/L. At this time, the patient became unable to take enteral feeds secondary to odynophagia. Esophagogastroduodenoscopy (EGD) was deferred, given his neutropenia. He was initiated on histamine-2 receptor inhibitor, a proton pump inhibitor, and empiric nystatin for presumed esophageal candidiasis. A bacterial culture grown from the contents of the patient's fistula grew *Actinotignum schaalii*. Infectious disease was consulted for treatment recommendations because of the rarity of this pathogen.^3^ His antibiotic treatment was de-escalated from ciprofloxacin to amoxicillin based on recommendations from the infectious disease department. His INR reached supratherapeutic levels. This elevation was hypothesized to be a presumed effect of ciprofloxacin on gastrointestinal gut flora, given decreased clearance with impaired kidney function. All anticoagulation was discontinued.

On day 10, the patient started to have black tarry stools, and the digital rectal examination showed black stool in the rectal vault. Vital signs were significant for heart rate 72 beats per minute, blood pressure 94/55 mm Hg, and SpO_2_ 92%. Gastroenterology was consulted, and the decision was made to perform an upper endoscopy to identify a source of bleeding. The patient was transferred to the Medical Intensive Care Unit for the management of supratherapeutic INR and melanotic stools. His laboratory test result values were remarkable for WBC 17.65 × 10^9^/L, hemoglobin 7.9 × 10^9^/L, hematocrit 24.9%, platelets 76, prothrombin time 69.8 seconds, INR 7.8, AST 13 U/L, and ALT 8 U/L. The patient was given 2 units of packed red blood cells, 8 units of fresh frozen plasma, and 10 mg of intravenous vitamin K. An EGD was performed on day 11 after correcting his INR (INR 1.5) and anemia. Endoscopy revealed diffuse circumferential black mucosa extending throughout the entire esophagus. Based on the EGD, a diagnosis of AEN was made. No biopsy was performed, given the risk of perforation. Prognosis and findings were discussed with the patient and his family, and the patient chose to be discharged to home on hospice.

## DISCUSSION

AEN, also known as black esophagus or Gurvits syndrome, is believed to be the manifestation of microvascular occlusion of the vessels supplying the esophagus and mucosal injury secondary to regurgitated stomach acid and pepsin. Black esophagus was first described in 1914 by Brekke et al in a patient with tuberculosis and sepsis, but the term “AEN” was not coined until 1990.^4,5^ The definition of AEN is now characterized by “striking circumferential black appearing friable esophageal mucosa extending from the gastroesophageal junction and involving various length of the organ proximally” on EGD or autopsy.^4^ AEN involves the distal esophagus in 97% of cases but can extend superiorly to include the proximal esophagus, such as in the case of our patient.^1^

The incidence of AEN is estimated to range from 0.01% to 0.28%.^1,2^ The prevalence of AEN is likely underestimated because of its lack of awareness, subclinical presentation, propensity for tissue regeneration, and the evanescence of the injury.^1^ Elderly men are affected disproportionately more than women (81%–88% based on 2 studies).^1,6^ A typical presentation of AEN is an upper gastrointestinal bleed with gastrointestinal symptoms (eg, dysphagia, epigastric pain) in the context of the shock of any etiology or another vascular event.^1,7^ Asymptomatic presentations of AEN have been reported too, such as in the case of a cancer patient during percutaneous endoscopic gastrostomy tube placement.^8^

The pathophysiology of AEN is poorly understood. The most well-supported theory describes a “two-hit” hypothesis surrounding vascular hypoperfusion and mucosal injury that results in basophilic necrosis of esophageal mucosa with a neutrophilic response.^1^ A rapid decrease in hemodynamic flow to the distal esophagus predisposes the esophagus to caustic injury from stomach contents.^1,9^ Neutrophilic enzymes occlude small vessels, resulting in gangrenous necrosis of the squamous epithelium and the activation of inflammatory cells. This occlusion may be the triggering mechanism in patients with microvascular conditions (eg, diabetes).^9^ AEN is classically discovered in critically ill patients with multiple comorbidities and hemodynamic instability or compromise. It has been attributed to hyperglycemia and gastric outlet obstruction or as a complication of bariatric surgery, substance abuse, hypoproteinemia and/or poor nutritional status, renal insufficiency, chronic respiratory disease, cardiac failure, cirrhosis, external compression of the esophagus, trauma, and major surgery.^7,10–17^ AEN has previously been reported in individuals with malignancy and in individuals with hypercoagulable states as well.^18^ Hyperglycemia has been described in as many as 90% of cases.

Previously, the diagnosis of AEN was supported with direct visualization rather than histopathological diagnosis.^9^ Recent evidence supports the use of a “diagnostic three times a day” of specific features on tissue biopsy (basophilic necrosis, pigment deposits, and vascular occlusion by fibrin thrombi in the esophageal mucosa) to diagnose AEN and to rule out alternative diagnoses.^9^ However, direct visualization of AEN is still considered to be the primary means of diagnosis because of the risk of intraoperative biopsy. The prognosis of AEN is poor. Mortality varies between 6% and 32%, likely secondary to complications from underlying comorbid illness and esophageal perforation.^1,7,9^ By increasing awareness surrounding the subclinical presentations of AEN, we hope to increase the rate of early intervention and reversal of this deadly condition.

## DISCLOSURES

Author contributions: E. Grisham wrote the manuscript, reviewed the literature and is the article guarantor. SA Khalaf edited the manuscript and reviewed the literature. V. Kuwajima edited the manuscript and approved the final manuscript.

Financial disclosure: None to report.

Informed consent was obtained for this case report.

## References

[1] Gurvits GE Cherian K Shami MN Black esophagus: New insights and multicenter international experience in 2014. Dig Dis Sci. 2015;60(2):444–53. 25297468 10.1007/s10620-014-3382-1

[2] Brar TS Helton R Zaidi Z Total parenteral nutrition successfully treating black esophagus secondary to hypovolemic shock. Case Rep Gastrointest Med. 2017;2017:4396870. 28702267 10.1155/2017/4396870 PMC5494091

[3] Lotte R Lotte L Ruimy R Actinotignum schaalii (formerly Actinobaculum schaalii): A newly recognized pathogen-review of the literature. Clin Microbiol Infect. 2016;22(1):28–36. 26577137 10.1016/j.cmi.2015.10.038

[4] Brekke A Nekrose av oesophagus. Med Rev. 1914(31):210–214.

[5] Goldenberg SP Wain SL Marignani P Acute necrotizing esophagitis. Gastroenterology. 1990;98(2):493–6. 2295407 10.1016/0016-5085(90)90844-q

[6] Gurvits GE Shapsis A Lau N Gualtieri N Robilotti JG Acute esophageal necrosis: A rare syndrome. J Gastroenterol. 2007;42(1):29–38. 10.1007/s00535-006-1974-z 17322991

[7] Ullah W Mehmood A Micaily I Khan MS Comprehensive review of acute oesophageal necrosis. BMJ Case Rep. 2019;12(2):227967. 10.1136/bcr-2018-227967 PMC6398709 30814100

[8] Rejchrt S Douda T Kopacova M Acute esophageal necrosis (black esophagus): Endoscopic and histopathologic appearance. Endoscopy. 2004;36(12):1133. 15578316 10.1055/s-2004-825971

[9] Jessurun J Cui I Aristi-Urista G Acute (gangrenous) esophageal necrosis (black esophagus): A rare form of injury with specific histologic features and diverse clinical associations with a common pathogenesis. Hum Pathol. 2019;87:44–50. 30825459 10.1016/j.humpath.2019.02.003

[10] Akaishi R Taniyama Y Sakurai T Acute esophageal necrosis with esophagus perforation treated by thoracoscopic subtotal esophagectomy and reconstructive surgery on a secondary esophageal stricture: A case report. Surg Case Rep. 2019;5(1):73. 31069560 10.1186/s40792-019-0636-3 PMC6506511

[11] Boersen GJ Geijteman ECT Schweitzer DH A man with a black oesophagus [in Dutch]. Ned Tijdschr Geneeskd. 2018:162:D187. 29676705

[12] Khan H Ahmed M Daoud M Philipose J Ahmed S Deeb L Acute esophageal necrosis: A view in the dark. Case Rep Gastroenterol. 2019;13(1):25–31. 31182940 10.1159/000496385 PMC6547274

[13] Kim DB Bowers S Thomas M Black and white esophagus: Rare presentations of severe esophageal ischemia. Semin Thorac Cardiovasc Surg. 2017;29(2):256–9. 28823340 10.1053/j.semtcvs.2017.01.006

[14] Lamers CR Mares WGN Bac DJ Black esophagus: A case series and literature review of acute esophageal necrosis. Scand J Gastroenterol. 2018;53(10–11):1421–4. 30353761 10.1080/00365521.2018.1513064

[15] McCarthy S Garland J Hensby-Bennett S Black esophagus (acute necrotizing esophagitis) and wischnewsky lesions in a death from diabetic ketoacidosis: A possible underlying mechanism. Am J Forensic Med Pathol. 2019;40(2):192–5. 10.1097/PAF.0000000000000463 30676335

[16] Moore C Matthews LR Danner O “Black esophagus” and gastric volvulus following slipped laparoscopic adjustable gastric band. Obes Surg. 2018;28(9):2941–8. 29905880 10.1007/s11695-018-3354-1

[17] Tse A Basu S Ali H Hamouda A Black necrotic oesophagus following the use of biodegradable stent for benign oesophageal stricture. J Surg Case Rep. 2015;2015(7):1–3. 10.1093/jscr/rjv072 PMC4491192 26142458

[18] Gurvits GE Black esophagus: Acute esophageal necrosis syndrome. World J Gastroenterol. 2010;16(26):3219–25. 20614476 10.3748/wjg.v16.i26.3219 PMC2900712

